# Extensive Pollen Flow but Few Pollen Donors and High Reproductive Variance in an Extremely Fragmented Landscape

**DOI:** 10.1371/journal.pone.0049012

**Published:** 2012-11-12

**Authors:** Rafael G. Albaladejo, Beatriz Guzmán, Santiago C. González-Martínez, Abelardo Aparicio

**Affiliations:** 1 Department of Plant Biology and Ecology, School of Pharmacy, University of Seville, Seville, Spain; 2 Department of Forest Ecology and Genetics, CIFOR-INIA, Madrid, Spain; CNR, Italy

## Abstract

Analysing pollen movement is a key to understanding the reproductive system of plant species and how it is influenced by the spatial distribution of potential mating partners in fragmented populations. Here we infer parameters related to levels of pollen movement and diversity of the effective pollen cloud for the wind-pollinated shrub *Pistacia lentiscus* across a highly disturbed landscape using microsatellite loci. Paternity analysis and the indirect KinDist and Mixed Effect Mating models were used to assess mating patterns, the pollen dispersal kernel, the effective number of males (*N_ep_*) and their relative individual fertility, as well as the existence of fine-scale spatial genetic structure in adult plants. All methods showed extensive pollen movement, with high rates of pollen flow from outside the study site (up to 73–93%), fat-tailed dispersal kernels and large average pollination distances (δ = 229–412 m). However, they also agreed in detecting very few pollen donors (*N_ep_* = 4.3–10.2) and a large variance in their reproductive success: 70% of males did not sire any offspring among the studied female plants and 5.5% of males were responsible for 50% of pollinations. Although we did not find reduced levels of genetic diversity, the adult population showed high levels of biparental inbreeding (14%) and strong spatial genetic structure (*S_p_* = 0.012), probably due to restricted seed dispersal and scarce safe sites for recruitment. Overall, limited seed dispersal and the scarcity of successful pollen donors can be contributing to generate local pedigrees and to increase inbreeding, the prelude of genetic impoverishment.

## Introduction

It is a general assertion that genetic drift compromises the evolutionary potential and long-term survival of fragmented populations throughout inbreeding depression and reduced response to selection [1], [2]. Consequently, determining in fragmentation studies the spatial scale at which gene flow is operating becomes essential since ecological and genetic isolation of populations may not coincide. Indeed, many empirical studies have shown, for example, that impacts of habitat fragmentation on plant mating systems are context-dependent and that habitat fragmentation can even increase pollination distances (i.e. gene dispersal) under specific circumstances [3], [4]. For the particular case of trees, which are candidates for long-distance dispersal both in space and time [5], the realised lack of concordance between theoretical expectations and empirical evidences raised the so-called ‘paradox of forest fragmentation genetics’ [6] and the claim for studies that focus gene dispersal and the precise spatial and temporal (offspring) scale that influences the species reproductive ecology [7].

Pollen flow is a main source of genetic variation among populations. Nevertheless, for small and fragmented populations not only the source of origin but the composition (i.e. diversity) of contributing pollen pool is equally important [7]. Hence, analysing pollen movements along with the diversity of the effective pollen cloud is a key to understanding shifts in plant mating systems associated to the particular spatial distribution of potential mating partners both within and among fragmented populations [8]. Factors such as the number of pollen donors contributing to the effective pollen cloud and the male reproductive variance (i. e. how different male reproductive success is across individuals) are highly relevant to delineate the genetic composition and performance of the next generation. High variance in the reproductive success of individual plants can enhance fragmentation effects by further reducing the number of partners available for mating [9].

*Pistacia lentiscus* L. (Anacardiaceae) is a common shrub or (more rarely) a small tree that is abundant in the Mediterranean sclerophyllous vegetation. Life history traits of this species resemble, to some extent, those of larger trees (i.e. long-lived, outcrosser, large pollen and seed crops, high potential for pollen and seed dispersal). Furthermore, the species can be found from almost continuous monospecific stands in forest understories to small clumps in open habitats and even isolated individuals; hence *P*. *lentiscus* constitutes a suited case study to analyse pollination biology, mating system and fine-scale population genetic structure in a fragmentation context. In a previous study performed in a large, dense and continuous stand of *P. lentiscus*, Albaladejo et al. [10] found that the species was characterized by (i) high genetic diversity of the effective pollen cloud, (ii) low levels of biparental inbreeding, and (iii) no signs of fine-scale spatial genetic structure (SGS), altogether attributable to extensive pollen (and seed) dispersal across the stand. But, from numerical simulations based on the observed data, the authors also foresaw that population attrition and clumping of individuals (a common situation for this and many other maquis’ species across the Mediterranean) could eventually reduce the number of males available for mating and result in a relatively high proportion of full-sibs within maternal progeny arrays (i.e. higher levels of correlated paternity).

In the present study, we focus on individual plants of *P. lentiscus* embedded in an intensively managed matrix. Our main goal is to assess the pollination biology of *P*. *lentiscus* by studying the pollination connectivity both in quantity and diversity across this highly disturbed landscape and compare the results with those obtained in continuous populations. Specifically, we address (i) the spatial patterns of pollen flow in the study site, (ii) the shape of the effective pollen dispersal distribution (i.e. the dispersal ‘kernel’) assessed by both direct and indirect methodologies, and (iii) the source diversity of successful mates (rates of biparental inbreeding, effective number of fathers and male reproductive variance). Further, we expect to find, (iv) lower levels of genetic diversity and a stronger pattern of spatial genetic structure of the adult population, and (v) higher levels of biparental inbreeding and within-mothers correlated paternity compared to continuous population due to noticeable long-term effects of fragmentation.

## Materials and Methods

### Study Species

*Pistacia lentiscus* is an evergreen long-lived shrub (very rarely a small tree up to 4 m in height) representative of the woody plant species in the Mediterranean. The species is dioecious (i.e. obligate outcrosser) and wind-pollinated, with a narrow flowering period in the studied area which spans from mid-March to late April (S. Nora, RG. Albaladejo and A. Aparicio, unpublished results). Fruits, which are small black one-seeded drupes, ripe from September to December when they are actively consumed by a wide array of small or mid-size birds, many of them migratory [11]. The species is not protected by law and permission to collect plant material was obtained from the Consejería de Medio Ambiente (Andalusian Regional Government).

### Study Landscape and Sampling

**Figure 1:**
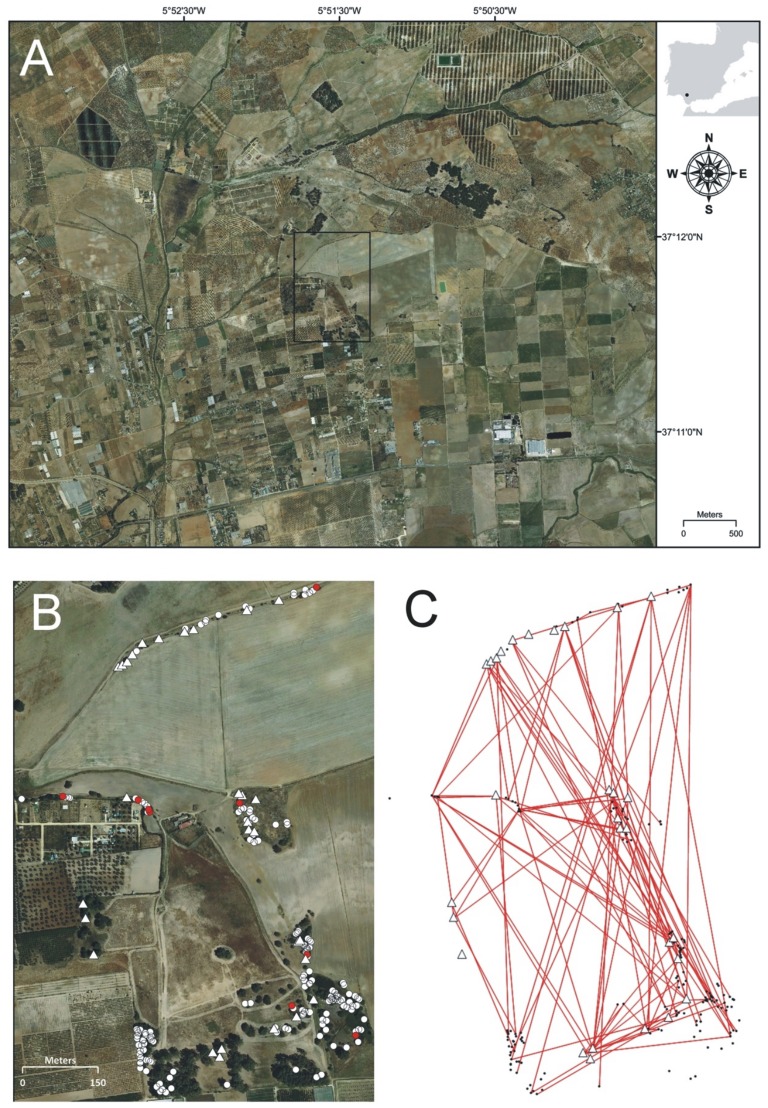
Aerial photography of the study site (black square) embedded in a highly anthropogenic area (A). Close up of the study site showing the 164 reproductive *Pistacia lentiscus* males (white circles) and the 29 mother plants (white triangles) sampled for mating and paternity analyses (B). Network of effective pollination events detected between males (dots) and females (triangles) in the paternity analysis (C). Red dots in panel B mark the position of males siring at least four seeds (see text for details).

The lower catchment of the Guadalquivir river (southern Spain) is a ‘relictual’-type agricultural landscape (*sensu* McIntyre & Hobbs [12]) characterized by very low habitat retention (natural or semi-natural woodlands covers only ca. 1% of its potential area), low connectivity between fragments and a high degree of anthropization [13]. Here, as study site we chose a rectangular area of ca. 70 ha (1000 × 700 m) (c. Utrera, 37°11’37”N, 05°51’31”W) embedded in a highly anthropogenic area devoted to cattle rising, cereal fields, vineyards, olive trees and scattered plantations of *Eucalyptus globulus* Labill. The study site itself is also a highly disturbed site (Fig. 1A, B) where only ca. 9.5 ha (<14% of the area) correspond to remnants of the original cork-oak (*Quercus suber* L.) vegetation and scattered clumps of *Pistacia lentiscus*, *Myrtus communis* L., *Quercus coccifera* L. and *Retama sphaerocarpa* (L.) Boiss. We chose this site because (1) the area is representative of the current vegetation found in highly-humanized Mediterranean landscapes, (2) the number of bushes of *P. lentiscus* was large enough to guarantee the representativeness of the study, (3) every individual plant could be accessed and identified with ease and (4) outside the study site no dense clump or stand of *P. lentiscus* exists in several km around (only scattered bushes can be found elsewhere). Where necessary, the landowners authorized access to private areas.

Within the study site we performed a consequential search and identified 514 reproductive plants of *P. lentiscus*, 350 females and 164 males (female-biased sex ratio, χ^2^ = 34.79, df = 1, *P*<0.001). In October 2007, we selected 29 mother plants distributed throughout the site (mean ± SD among-mother distance 425 m ±237 m, range 5–914 m; Fig. 1B) from which we collected a mean (± SD) number of fully mature black fruits of 69.6±59.3 (range 36–306) directly from all over the plant crowns. Overall, we collected 2014 fruits and the existence of a viable seed inside was verified by sinking them in water. Because many black fruits also contained aborted embryos, the final progeny array consisted of 690 seeds available for genetic analysis, with a mean (± SD) number of seeds per mother plant of 24 (±4). Young leaves of all 164 *P*. *lentiscus* reproductive males in the study site and the 29 mother plants were also collected and kept dried in silica-gel until DNA isolation. All the studied plants were georeferenced.

### DNA Isolation and Genotyping

We isolated total genomic DNA from seeds and adult plants with the Invisorb DNA Plant HTS 96 Kit (Invitek, Berlin-Buch, Germany) according to the manufacturer’s protocol. We amplified seven unlinked polymorphic nuclear microsatellite loci following amplification conditions and PCR cycle profiles provided in Albaladejo et al. [14]. Amplified products were fluorescently labelled, with 6-FAM, NED, VIC or PET, and analysed on an ABI 3730 DNA Analyzer (PE Applied Biosystems, Foster City, CA, US). Fingerprint profiles were automatically scored with the software GeneMapper v.3.7 (PE Applied Biosystems, Foster City, CA, US) and visually inspected for corrections. All seeds were successfully genotyped for at least five loci each.

### Pollen Flow and Shape of the Pollen Dispersal Distribution

The pollen movement among individual plants within the study site was assessed using paternity analyses to assign each seed to its most-likely father by the maximum likelihood approach implemented in Famoz [15]. Confidence in paternity assignments was obtained by comparing the distribution of the logarithm of the odd ratios (*LOD* scores) of the most-likely fathers of 50000 synthetic seeds with their father chosen among the 164 potential males, to the distribution of *LOD* scores of the most-likely father of 50000 seeds whose paternal genotype was randomly generated according to reference population allele frequencies. In wind-pollinated species, levels of pollen immigration can be high [16], so extracting allele frequencies from the local male population can be misleading since the actual breeding population might be much larger. Then, we extracted the reference allele frequencies from the bigger sample of 690 seeds, after subtracting the maternal contribution following Hardy et al. [17]. Threshold value for rejecting a candidate male as a true father was *TF* = 5.50 (i.e. the value at the intersection of the two *LOD* score distributions [15]. Since we are mostly interested in describing patterns of pollen flow rather than minimizing Type I errors in the assigning of seeds to specific fathers, the genotyping error was set to zero to avoid increasing assignment error [18]. The inbreeding coefficient (*F*) was set to 0.152, the value estimated for the adult population (see Results). After running the analysis we placed each analysed seed into one of the following three categories: (i) no compatible father in the study site (i.e. the minimum bound of incoming pollen flow rate), (ii) at least one compatible father but with a *LOD* score < *TF*, and (iii) one or multiple candidate fathers within the site with a *LOD* score > *TF*. In the latter case, we assigned paternity to the spatially closest male. We believe that the potential bias introduced by this procedure is negligible because most ties in our dataset occurred between genetically related neighbouring males.

To check whether mating success was a mere function of the spatial position of males and females (i.e. ‘flat’ dispersal kernel), we compared the observed frequency distribution of mating events with its potential distribution (considering all 4756 possible mating events between the 164 male and 29 female plants) using a Kolmogorov-Smirnov test. We also evaluated the directionality (i.e. isotropic vs. anisotropic) of this distribution [19] with regard to the direction of the prevalent winds during the flowering period (15th March to 31st April). Wind data were taken from the nearest climatic station (‘Los Palacios y Villafranca’; 37°10'49''N, 05°56'15''W) at similar altitude and climatic conditions (data available at http://www.juntadeandalucia.es/agriculturaypesca/ifapa). Directional frequency histograms of the distribution of observed and potential mating, and prevailing wind direction were compared through Watson’s *U^2^*-test using the R package *circular* [20].

The shape of the effective pollen dispersal curve was characterized using two different indirect methods. Firstly, we use the KinDist approach [21], which is based on the expected decay of correlated paternity among maternal pairs with distance, and provides minimum squared-error estimates for the scale (*a*) and shape (*b*) parameters of the power-exponential dispersal function as well as the average effective pollination distance (δ) derived from it [22]. The parameter *b* controls the tail of the distribution so that *b* <1 provides fat-tailed dispersal functions (which can better account for long-distance pollen dispersal events) while *b* >1 provides thin-tailed functions. In our case, we first checked that among-mothers correlated paternity significantly decreased with (log) distance (Pearson *r* = −0.125; *P* = 0.012, Figure S1) and then set a threshold distance for unrelated pollen clouds of 300 m. This analysis was carried out using Poldisp v.1.0c software [23].

Secondly, we used the Bayesian Mixed Effect Mating model [9] which estimates not only the shape parameter of the power-exponential function, but also jointly assesses selfing (*s*) and immigration rates (*m*), the ratio between observed and effective male density (*d_obs_/d_ep_*) and, remarkably, the relative individual fecundity of each male (see below) in the study site. We used the following minimum and maximum values as prior information: *d_obs_/d_ep_* = 1, 150; δ = 50, 1500; *b* = 0.1, 10; and *m* = 0.4, 0.95. Because *P. lentiscus* is dioecious, we set to zero the prior values for *s*. Since the analysis requires from reference outside allele frequencies for the calculation of the immigration rate (see eq. 6 in Klein et al. [9]), we used the allele frequencies obtained from the set of 569 seeds for which no fathers within the study site could be confidently assigned (*LOD* score < *TF*) after the Famoz analysis. The analysis was run for 100000 iterations plus an initial burn-in period of 20000 iterations. We also included a covariate in the analysis which reports on the local environment where each male was located in the study site (similar to the neighbourhood density in [24]) following three categories of decreasing neighbourhood density: (1) under a dense canopy of cork oak or eucalyptus trees, (2) in sparse shrublands with no trees in the canopy, and (3) in linear hedges. This analysis was performed with the software MEMM 1.1 [9].

### Mating System, Effective Density of Pollen Donors and Male Reproductive Variance

Biparental inbreeding (i.e. the mating between close relatives; *t_m_*-*t_s_*) was calculated with the software MLTR 3.3 [25] from the multilocus (*t_m_*) and single locus (*t_s_*) outcrossing rates estimated through the Newton-Raphson algorithm. We assessed *t_m_*-*t_s_* statistical significance by comparing 1000 bootstrap values of *t_m_* vs. *t_s_* by means of Student’s paired *t*-tests. Bootstrap replicates were done with resampling of families. Within-mother correlated paternity (*r_p_*) was calculated as twice the average pairwise kinship coefficient *F_ij_* [26] between paternal genes of seed pairs. To check whether the composition of progeny arrays, measured as the proportion of full sibs within maternal families, was a direct consequence of the local environment of females, we correlated individual within-mother *r_p_* values against the distance to the nearest male and to the nearest three and five males, respectively. The effective number of pollen donors for the sampled mothers in the study site (*N_ep_*) was deduced as *r_p_* ^−1^. We compared this result with the *N_ep_* value derived from the mean value of the *d_obs_/d_e_* probability density function obtained from the MEMM analysis.

Male reproductive success in the study site was estimated directly from the paternity analysis with Famoz and with the MEMM software. In the Mixed Effect Mating model, male relative fecundities are modelled as random effects under a Bayesian framework. We ran two analyses assuming individual male fecundity to follow either a log-normal or a gamma distribution [9].

### Genetic Diversity and Spatial Genetic Structure (SGS) of Adults

For the adult plants (29 females and 164 males), we also calculated single-locus descriptive diversity parameters using Fstat v.2.9.3.2 [27], including the number of alleles (*A*), the expected heterozygosity (*H_e_*) and the inbreeding coefficient (*f*). Departure from Hardy-Weinberg (HW) equilibrium was assessed by means of a Markov Chain-based exact test using Genepop v.4 [28] under default settings (10000 dememorization steps, 20 batches and 5000 iterations per batch). Null allele frequencies were also estimated with Genepop v.4.

We analysed the existence of SGS in the adults by regressing the pairwise kinship coefficients (*F_ij_*) between individuals on the logarithm of the spatial distance as corresponding to a bidimensional space [29]. The significance of SGS was obtained by comparing the observed regression slope with that obtained from randomly permuting (1000 times) the spatial location of the adult individuals [30]. Patterns of SGS were visualized by constructing a correlogram plotting the average *F_ij_* values into distance classes. To allow a direct comparison with the continuous population studied by Albaladejo et al. [10] we restricted the analysis to same maximal distance (460 m) and constructed the same 10 distance classes. Approximate 95% confidence intervals (CI) were calculated for the *F_ij_* values as twice the standard error (SE) obtained by jackniffing over loci. The intensity of the SGS was also quantified by the *S_p_* statistic, a dimensionless parameter useful to perform comparisons between species and populations [30]. *S_p_* is calculated as *b*-log/(*F*_(1)_−1), where *F*_(1)_ is the mean *F_ij_* among individuals pairs in the first distance class and *b*-log is the slope of the correlogram. All SGS analyses were performed with the software SPAGeDi v.1.2 [31].

## Results

### Pollen Flow and Shape of the Pollen Dispersal Distribution

The cumulated exclusion probability (when the mother is known) for the seven microsatellite loci used was 0.995, supporting the suitability of our dataset to conduct a paternity analysis (see Table 1). The estimated frequencies of null alleles were on average low, only moderate (≥0.150) for the loci *Pislen 114* and *Pislen R05* (Table 1). Out of the 690 genotyped seeds, 121 (17.5%) had at least one candidate father with a *LOD* score > *TF*. Additionally, 63 seeds (9.1%) had at least a compatible father but with a *LOD* score below the threshold required for a confident assignment. This result means that a minimum of 73.3% and a maximum of 82.4% of the seeds were sired by fathers located outside our study site.

**Table 1:** Genetic diversity parameters for *Pistacia lentiscus* adult plants genotyped at seven microsatellite loci.

Locus	A	H_e_	f	Null	Exc
Pislen 21	14	0.820	0.204***	0.090	0.684
Pislen 114	6	0.704	−0.067**	0.178	0.497
Pislen 333	18	0.883	0.249***	0.112	0.745
Pislen 501	10	0.774	0.210***	0.097	0.587
Pislen 510	15	0.779	0.189***	0.085	0.609
Pislen 526	3	0.432	0.010^ns^	0.006	0.180
Pislen R05	3	0.212	0.205**	0.150	0.079
Overall	69	0.658	0.152***	–	0.995

** *P*<0.01;

*** *P*<0.001;

ns not significant.

Number of alleles (*A*), expected heterozigosity (*H_e_*), Weir and Cockerham’s (1984) inbreeding coefficient (*f*), null allele frequency (*Null*) and exclusion probability for paternity analysis (*Exc*).

**Figure 2:**
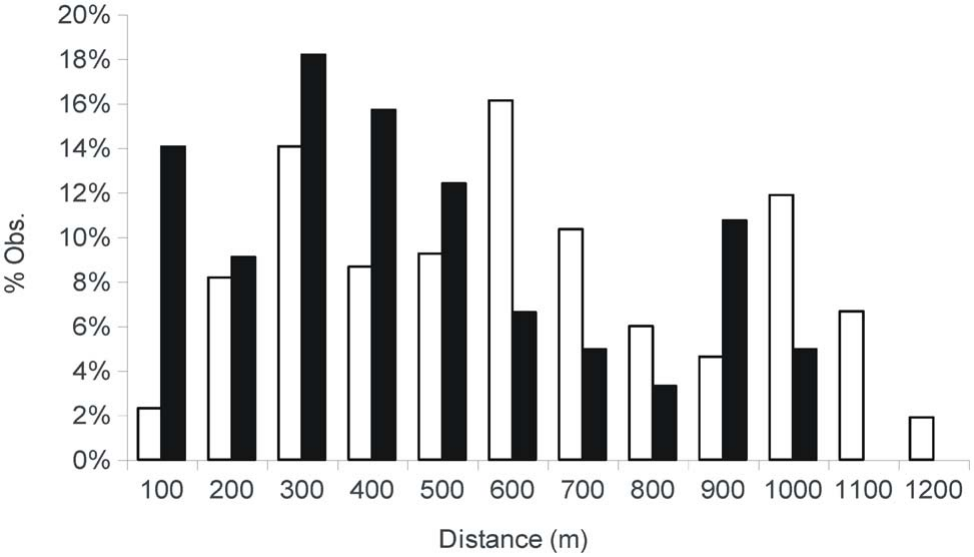
Frequency histograms of effective pollination distances (black bars) estimated via paternity analysis and pairwise distances between males and the sampled mother plants (white bars).

The paternity analysis also detected high connectivity within the study area (Fig. 1C). The mean (± SD) dispersal distance for the 121 confidently assigned male-offspring pairs was 412 m (±276) (range 9–970 m) with about 50% of the seeds being sired by fathers located more than 370 m apart. The success of mating events was not a mere function of the spatial distribution of males and females since the observed mating frequency distribution was significantly different from the potential distribution (K-S *d* = 0.280; *P*<0.001). In spite of the large distance for the observed matings, these occurred at distances shorter than expected (Fig. 2). Thus, siring events were not produced at random with regard to among-mates distance.

**Figure 3:**
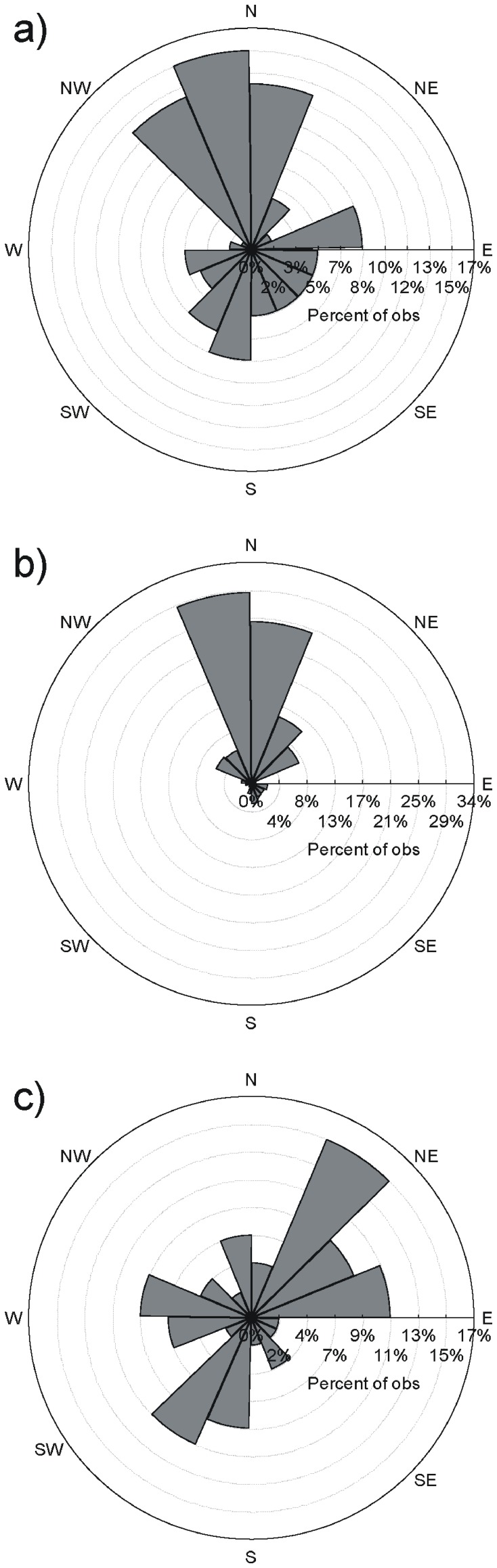
Wind rose percentage frequency histograms of (A) the direction of mating events detected in the paternity analysis, (B) the direction of random potential mating events (i.e. just conditioned by the spatial location of males and females), and (C) the direction of winds during the flowering season.

Most pollination events took place in the S-N direction (Fig. 3A) according to the relative spatial arrangement of males and females sampled in the plot (Fig. 3B). During the blooming phase, prevailing winds occurred in N-E and S-W directions (Fig. 3C). The observed directional distribution of mating events was significantly different from both the expected random distribution considering spatial position of males and females alone (*U^2^*-test = 0.432; *P*<0.001) and the one of prevailing winds during the flowering period (*U^2^*-test = 0.252; *P*<0.05). Interestingly, the observed distribution was a somewhat combined distribution considering both the spatial position and wind direction.

The KinDist analysis successfully fitted a power-exponential function to the data and provided point estimates for the scale and shape parameters of *a* = 2.8 10^−4^ and *b* = 0.16, as well as an average effective dispersal distance of δ = 268.5 m. Normal, geometric or exponential functions showed a poorer fit based on the least-square residuals (results not shown).

The mean conditional log-likelihood for the two runs with MEMM, either assuming a log-normal or gamma distribution for individual male fecundity, were −8145 and −8138 respectively; an approximate Bayes factor, computed as the ratio of the estimated likelihoods, strongly supported the model with the gamma distribution (BF ≈ 1097; values above 100 are usually considered as decisive to support one model against the alternative [32]). Consequently, we show here only the results of the second run (gamma distribution). Regarding the fitted function, the Mixed Effects Mating model provided similar results to the KinDist approach, with mean values (and 95% credibility intervals) for the posterior distribution of *b* = 0.19 (0.10–0.46) and δ = 229 m (52–1069 m). Additionally, the immigration rate estimated by MEMM was very high, *m* = 0.937 (0.915–0.949), which supports the results of the direct paternity analysis.

### Mating System, Effective Density of Pollen Donors and Male Reproductive Variance

In the study site, we found a highly significant difference between multilocus and single-locus outcrossing rates (*t_m_* = 0.999 vs. *t_s_* = 0.856; Student’s *t* = 94.466, *P*<0.001), indicating that about 14% of matings occurred between genetically related plants. Individual within mother correlated paternity showed a significant negative correlation with the (log)distance to the nearest male (Pearson *r* = −0.382, *P* = 0.041) but this trend vanished and became non-significant when referred to the average distance to the nearest three and five males (*r* = −0.308, *P* = 0.104 and *r* = −0.297, *P* = 0.118). The average within mother correlated paternity calculated from kinship coefficients was also high (*r_p_* = 0.231) indicating that in average about 23% of seeds in a given mother plant were sired by the same father. This value translates into an effective number of fathers *N_ep_* = 4.3. The Mixed Effects Mating model provided a posterior mean value (95% credibility interval) for the ratio *d_obs_/d_e_* = 16 (2.7–45) which corresponded to a *N_ep_* value of 10.2 (3.6–60.7) according to the observed census of males (*N_obs_* = 164) in the study site.

**Figure 4:**
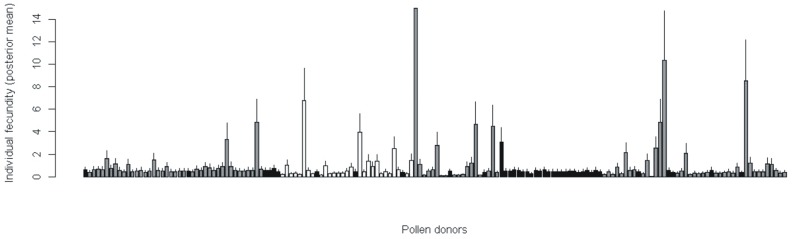
Individual relative fecundities of the 164 *Pistacia lentiscus* male plants estimated using Klein’s Mixed Effect Mating model. Different shades refer to male plants occurring in different landscape typologies: dense tree canopy (black), sparse shrublands (grey), and linear hedges (white). Bars represent the 95% credibility intervals.

Based on the paternity analysis, male reproductive success was highly leptokurtic since 114 out the 164 males (70%) did not sire any seed in any of the 29 females sampled within the study area. Only nine fathers (5.5%) were responsible for nearly 50% of the assignments (red dots in Fig. 1B), each of them siring four or more seeds and one father siring 15 seeds. These results were concordant with those obtained from the MEMM analysis, estimated jointly with the dispersal parameters, which revealed very few males with a very high fecundity related to a majority of males with a poor reproductive contribution. Fecund males were spatially located in sparse shrublands and linear hedges while none occurred under a dense tree canopy (Fig. 4).

**Genetic diversity and fine-scale SGS of adults.**

Genetic diversity of *P. lentiscus* was relatively high (*A* = 69, *H_e_* = 0.658). However, most loci showed a significant excess of homozygotes (Table 1).

**Figure 5:**
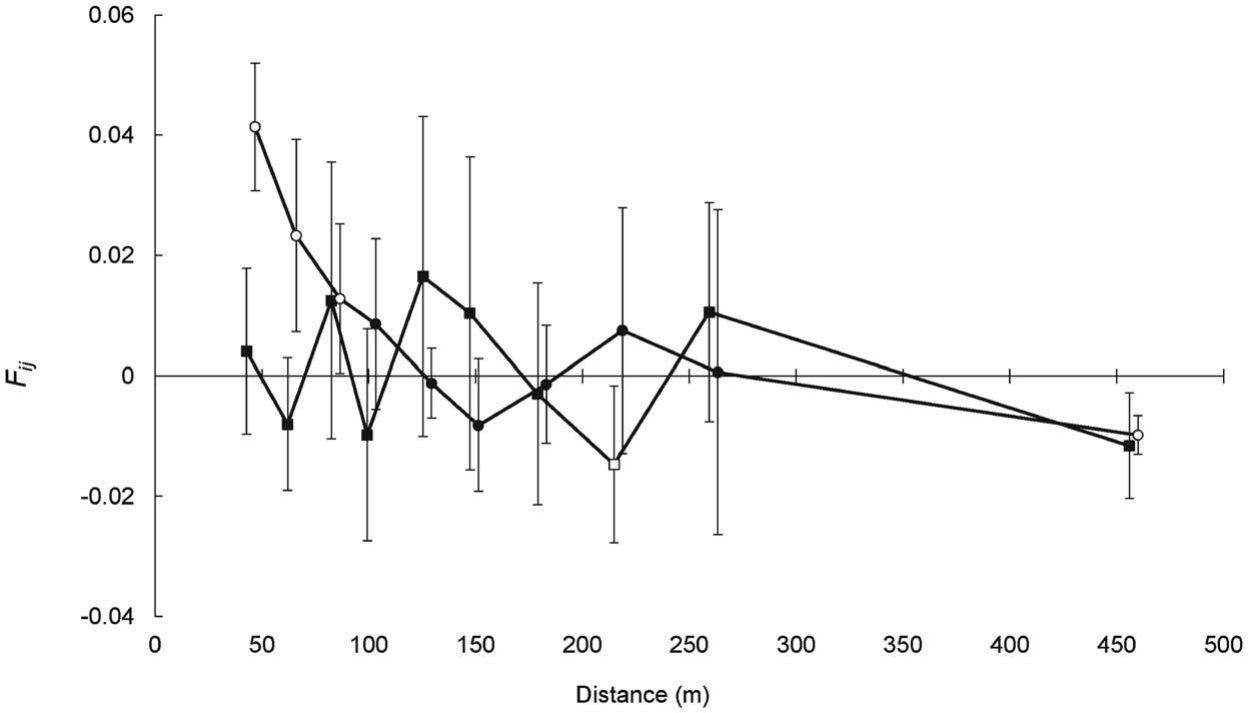
Average pairwise kinship coefficients (*F_ij_*) for adult plants plotted against spatial distance in the studied fragmented landscape (circles) and in a previously-studied large continuous population (squares; reanalysed from [**10**]). Error bars represent approximate 95% confidence intervals and empty symbols mean they are significantly different from the null hypothesis of no spatial structure assessed trough permutation procedures (1000 permutations). Symbols have been slightly scrolled to facilitate visualization.

The overall slope of the correlation between pairwise kinship and (log) distance was highly significant (*b*-log = −0.0117; *P*<0.001) indicating that genetically related adult individuals were aggregated across the space. The correlogram revealed a sharp decline in the kinship coefficients with distance, with significant positive average *F_ij_* in the first three distance classes, which extended through a distance of approximately 85 m (Fig. 5). The calculated value for the *S_p_* statistic was 0.0122.

## Discussion

Long term viability of populations relies on maintaining adequate levels of genetic variation and gene flow [1]. Consequently, a considerable research activity on the mechanisms of dispersal of pollen and seeds and its genetic and demographic consequences has been developed in recent decades [33]. From a conservation and landscape genetics perspective, analyzing patterns of pollen dispersal in disparate landscapes should allow visualizing those shifts in the pollination biology patterns of species that could ultimately foster inbreeding depression, genetic impoverishment and population divergence.

### Pollen Flow

Our analyses provided compelling evidence that pollen movement in the studied area was extensive, not only within the study site, but also from outside: between 73–93% (depending on the method) of the sampled seeds were sired by incoming pollen flow. Admittedly, we ignore the actual location of the male plants that contributed so remarkably as pollen donors, but we believe that they must probably be located in privileged positions (see below) in the vicinity of the study site, despite only scattered bushes and very small clumps of *P. lentiscus* can be found in the surroundings. The closest relatively large population of the species is located ten kilometres eastwards but can probably be ruled out as a source of pollen in our data set given the estimated low numbers of effective pollen donors found for single mothers. Indeed, if incoming pollen was originated in a large population, then, many different fathers would be expected to contribute to single mothers, increasing *N_ep_* estimates. Therefore, despite the study site appears remote and disconnected, our results highlight the cohesive role that neglected small vegetation patches or isolated individuals may play in highly fragmented landscapes [34], particularly if high effective pollen dispersal is inherent to the reproductive biology of the species.

In agreement, both the direct and indirect methods that we used indicated large average pollination distances, although the estimates varied somehow across the methods. The paternity analysis, which is constricted by the precise spatial distribution of sampled female plants (given that not all female plants were sampled), provided an estimate of about 400 m, well above the mean dispersal distance inferred through direct methods for wind-pollinated species [35]. Both indirect mating models (KinDist and Klein’s mixed effect model) showed an average dispersal distance above 200 m, which is also a comparatively high estimate (e.g. [36]). This latter is perhaps a more realistic estimate because the fitting of dispersal kernels provides a more general picture of pollen dispersal patterns, as if pollen could land everywhere [18], [34]. A direct comparison with the pollen dispersal kernel in a previously-studied continuous population [10] was not possible due to the weak genetic structure of the effective pollen cloud in that case, which did not allow for convergence of model fitting algorithms. However, this result suggests pollen dispersal kernels in unfragmented situations to be even flatter than in fragmented ones.

Although we have shown extensive pollen flow in this fragmented landscape there is also an important component of restricted pollen dispersal at local scales. Below 500 m, the paternity analysis reveals more matings than expected at random (see Fig. 2), which support the view of pollen dispersal in wind-pollinated plants to be characterized by a fraction of short distance pollinations and another fraction of pollinations occurring at large distances enlarging the tail of the pollen dispersal distribution [35].

Assessing the directional distribution of successful matings is also important because it may determinate the number of sires available for female plants. Our sampling scheme resulted in heterogeneity in the directional distribution of males and the sampled females (with northwards matings being clearly favoured), which surely is responsible for the high proportion of matings in the S-N direction we detected (see Figs. 3A & B). Interestingly, we also detected relatively high levels of successful mating events in the third (S-W) quadrant, probably influenced by the S-W prevailing winds during the blooming period (see Figs. 3A & C). Many studies have not detected effects of regional winds on pollen dispersal patterns (e.g. [37], [38]). This discordance might indicate lack of potential males in favourable wind directions and/or that pollen dispersal is influenced by localized wind patterns, only detectable by placing meteorological stations within the study plot (an issue, however, that seems not relevant in the flatlands of our study area). In addition, detecting clear anisotropic pollen dispersal patterns in the spread of pollen clouds may be also a technical issue since it requires a greater genotyping effort (here 29 mother plants and 690 offspring) than usual [19].

### Mating System and Male Reproductive Success

One aspect of the reproductive ecology of plants that becomes crucial in fragmented populations is correlated paternity (the diversity of fathers siring progeny in a mother) because where spatial connectivity and the availability of suitable places for seed arrival and seedlings establishment is reduced, progeny arrays composed of full-sibs can perform poorly compared to those composed of half- or unrelated sibs [39]. Moreover, it is expected that population attrition and the spatial clumping of pollen sources increases the proportion of full-sibs in the annual crop of a female plant [40], an outcome that Albaladejo et al. [10] predicted for this species through numerical simulations. Therefore, it is very important to stress that in our study site, we have found about six-fold higher values of correlated paternity compared to the large, dense and continuous stand of the same species studied by Albaladejo et al. [10] (23% vs. 3–8%, respectively).

Besides high correlated paternity, we have also found a strong variance in male relative fecundity since just a few males have copped a high proportion of all mating events. To explain this unbalance in male contributions several reasons have been adduced, among them differential quantity or quality of the pollen released [24], lack of synchrony in the blooming period [41] and/or density depended effects. In our case, males that sired a high number of offspring were located in areas of low vegetation density (see red dots in Fig. 1) on sparse shrublands or hedges, but none under a dense tree canopy (Fig. 2). In wind-pollinated species pollen grains released at the edge of vegetation patches have less aerodynamic impediments to travel than those released within closed stands [42]. In agreement with this observation, density dependent factors and the father ecological neighborhood seem to be determinant in our study site; nevertheless, we cannot rule out some effects from blooming synchrony and pollen quality acting in concert.

We found significant levels of inbreeding in all but one locus, which in this dioecious species can be explained by significant levels of biparental inbreeding. A relevant impact of null alleles was ruled out because their estimated frequency was in average low and because significant inbreeding was also detected in the same population with other nuclear markers [43].

Biparental inbreeding accounted for a moderate portion (14%) of the mating events, being probably a straight consequence of the existence of significant SGS (see below) and to the fact that mating were more likely at short distances than at random (discussed above). Further, the proportion of full-sibs within mother progeny arrays is significantly higher as the distance to the nearest male decreases which suggest some degree of monopolization of some females by their closest male pollen cloud. However, this influence is spatially very restricted since only the relationship with the distance to the nearest male was significant. This negative relationship (albeit weaker) was also found in a previously-studied continuous population but only in one of the studied seasons [10].

### Genetic Variation and Fine-scale SGS of Adults

Despite the extreme disturbance of the studied landscape, genetic diversity of *P. lentiscus* was relatively high and similar to that of other Mediterranean woody plants, either in fragmented populations or not [2], including that obtained in continuous populations of the same species [10]. Individual longevity, obligate outcrossing, high rates of pollen gene flow and low population genetic differentiation at the regional scale [43] seem to make this species resistant to an immediate loss of allelic diversity, similarly to other long-lived woody plants [44].

Finally, the *S_p_* value (0.0122) is indicative of a strong pattern of SGS, since it is roughly twice the typical for wind-pollinated (0.0064) or animal-dispersed (0.0088) plant species [30], and more important, it is more than five times higher than the one found for the same species in a previously-studied large and continuous population where no significant pattern of SGS was detected (*S_p_* = 0.0022; recomputed from [10]). Besides the scarcity of successful pollen donors, patterns of spatial genetic structure are also dependent on the effectiveness of seed dispersal. Despite a wide array of bird species feeds on fruits of *P. lentiscus* [11], in our particular study system the ecological conditions are probably limiting seed dispersal and recruitment (i.e. if there are very few suitable safe sites, dispersers would not reach distant safe sites, and most seeds would be dispersed from only a few mother plants [45]). Extreme anthropogenic pressure can restrict safe microsites for recruitment just below the canopy of mother plants, fostering the formation of genetic pedigrees and maternal and paternal correlations (i. e. the probability of sharing parental plants) in the seed rain.

### Conclusions

The real impact of anthropization on the viability and performance of natural plant populations is still controversial. So far, ideal experimental designs under a fragmentation genetics perspective are very difficult to meet in nature. Moreover, to assess the actual scale of gene dispersal (pollen and/or seed) within and among plant populations (especially for trees and long-lived shrubs) become essential to reconcile the theoretical and empirical evidence [7].

Keeping this idea in mind, this is one of the scarce studies devoted to assess pollen dispersal and the shifts imposed by severe anthropization in the mating system of a long-lived woody plant species. Specifically, our study illustrates the paramount importance of small clumps or isolated individual plants in keeping genetic connectivity even in, at first glance, remote and isolated populations. Indeed, we have found genetic diversity (number of alleles and heterozygosity) at levels that, in the light of the ecological characteristics of the study site, were unexpected.

Nevertheless, we also found that most male plants did not sire a single seed from the studied female plant progenies and that the mating system of *P. lentiscus* was severely impacted by fragmentation: compared to a large and continuous population [10], the highly-disturbed population studied here had increased biparental inbreeding, increased correlated paternity (and decreased number of pollen donors) and highly significant spatial genetic structure. Not only the pollen cloud perceived by females is less diverse, but also, due probably to the scarcity of suited places for recruitment, the species is experiencing the formation of local pedigrees and increased inbreeding, the prelude of genetic impoverishment.

## Supporting Information

Figure S1 Correlation between among mother correlated paternity (i.e. proportion of half-sib among mothers) against the distance (at logarithmic scale) among mothers pairs.
(PDF)
