# Supplementary material for: Extensive Pollen Flow but Few Pollen Donors and High Reproductive Variance in an Extremely Fragmented Landscape
Source: PLoS One. 2012 Nov 12;7(11):e49012. doi: 10.1371/journal.pone.0049012 (PMC3495779; doi:10.1371/journal.pone.0049012)

Figure S1. Correlation between among mother correlated paternity (i.e. proportion of half-sib among mothers) against the distance (at logarithmic scale) among mothers pairs.

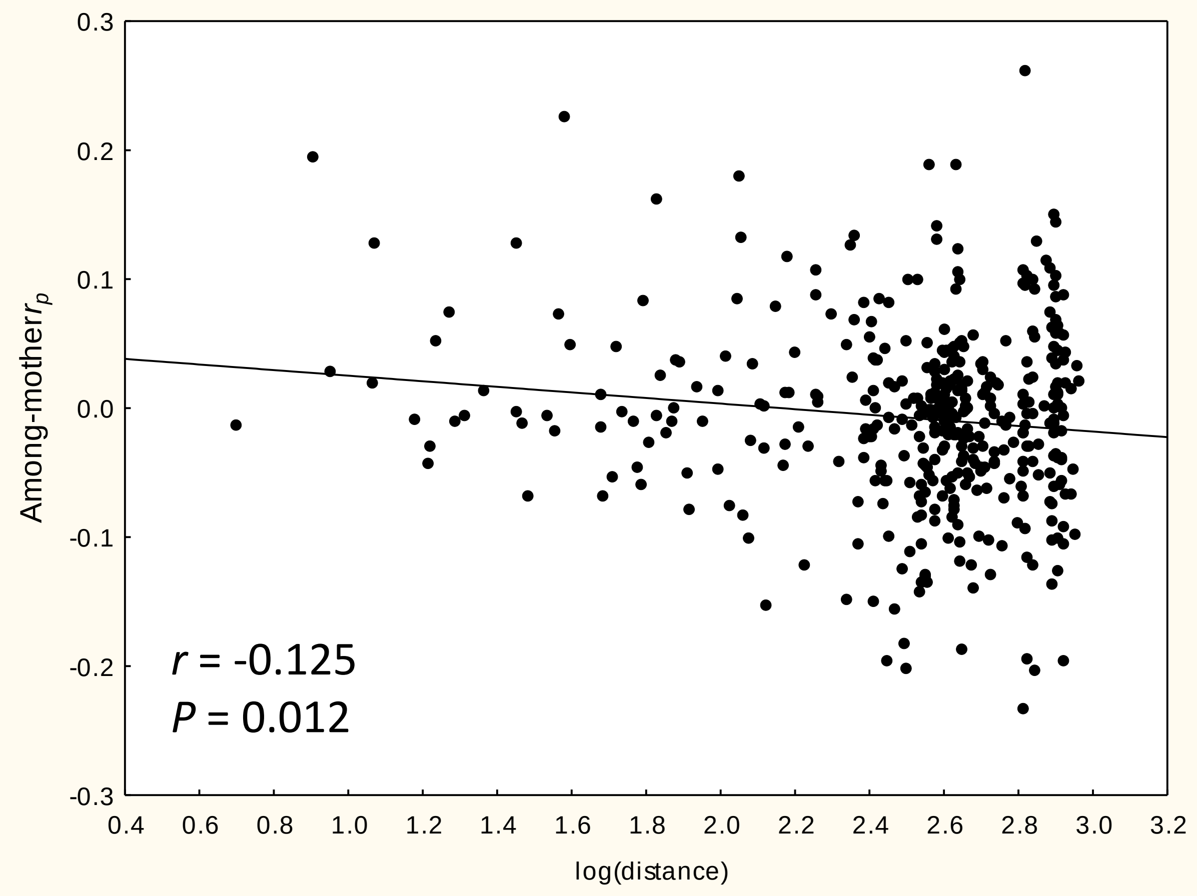

Supplement: Figure S1 — Correlation between among mother correlated paternity (i.e. proportion of half-sib among mothers) against the distance (at logarithmic scale) among mothers pairs. (PDF) [file pone.0049012.s001.pdf]
